# Combination of Ablation and Immunotherapy for Hepatocellular Carcinoma: Where We Are and Where to Go

**DOI:** 10.3389/fimmu.2021.792781

**Published:** 2021-12-15

**Authors:** Kunpeng Wang, Cong Wang, Hao Jiang, Yaqiong Zhang, Weidong Lin, Jinggang Mo, Chong Jin

**Affiliations:** ^1^ Department of General Surgery, Taizhou Central Hospital (Taizhou University Hospital), Taizhou, China; ^2^ Department of General Surgery, Second Xiangya Hospital, Central South University, Changsha, China; ^3^ Department of Clinical Laboratory, Taizhou Central Hospital (Taizhou University Hospital), Taizhou, China

**Keywords:** hepatocellular carcinoma, ablation, immunotherapy, synergistic therapy, multifunctional nanoplatform, nanomedicine

## Abstract

Hepatocellular carcinoma (HCC) is the third leading cause of cancer-related deaths worldwide and is increasing in incidence. Local ablative therapy plays a leading role in HCC treatment. Radiofrequency (RFA) is one of the first-line therapies for early local ablation. Other local ablation techniques (e.g., microwave ablation, cryoablation, irreversible electroporation, phototherapy.) have been extensively explored in clinical trials or cell/animal studies but have not yet been established as a standard treatment or applied clinically. On the one hand, single treatment may not meet the needs. On the other hand, ablative therapy can stimulate local and systemic immune effects. The combination strategy of immunotherapy and ablation is reasonable. In this review, we briefly summarized the current status and progress of ablation and immunotherapy for HCC. The immune effects of local ablation and the strategies of combination therapy, especially synergistic strategies based on biomedical materials, were discussed. This review is hoped to provide references for future researches on ablative immunotherapy to arrive to a promising new era of HCC treatment.

## Introduction

Primary liver cancer is one of the most common malignant tumors in the digestive system and the third leading cause of cancer-related deaths (1). Hepatocellular carcinoma (HCC), which comprises ~90% of cases, is the most common type of primary liver cancer. The management of HCC lies on the Barcelona Clinic Liver Cancer (BCLC) staging system. Most clinical practices guidelines recommend resection, thermal ablation and transplantation for patients with early HCC (BCLC 0, A), whereas transarterial chemoembolization (TACE) and systemic therapies are preferred for patients with intermediate (BCLC B) and advanced (BCLC C) HCCs, respectively (2–5). Surgical resection and transplantation could offer the best chance for a cure in early HCC, but not all patients with early-stage HCC, especially those with cirrhosis, benefit from these treatments. Liver function and portal hypertension are the fatal selection criteria of resection, because 80%-90% HCCs develop from cirrhosis (6). Moreover, the recurrence rate after HCC resection reaches as high as 68% (7). Scientists and surgeons have exerted much effort into the removal of tumors (8). However, this task is still an insurmountable mountain, because HCC cannot be considered a local disease even in the early stage. The outcomes of liver transplantation are superior to that of hepatic resection. However, organ shortage, long waiting time, and high cost are deterred, except for the strict transplantation indication. Locoregional ablative therapy including radiofrequency ablation (RFA) and microwave ablation (MWA), is a potentially curative strategy for early HCC, coming into sight. The advent of the genomic era, as well as the increase in the understanding of the role of immunity in HCC progression, support targeted therapy and immunotherapy. The combination of ablative therapy and immunotherapy has been a subject of recent clinical and basic researches. Herein, we summarized ablative therapy and immunotherapy for HCC, discussed their synergistic anti-tumor effects, and envisaged the current trends and future prospects of their combination.

## Ablation Therapy

**Figure 1:**
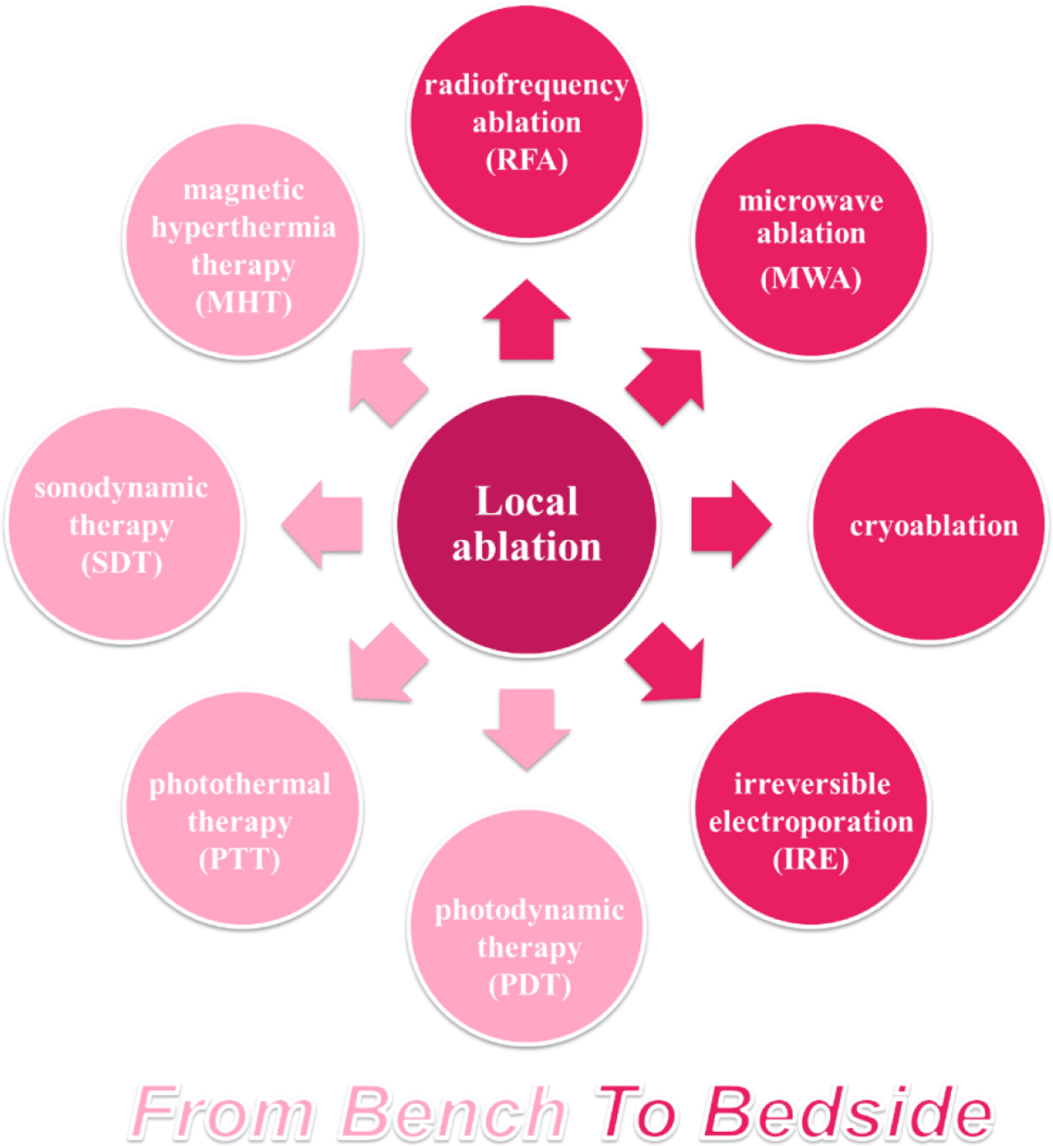
Overview of ablative techniques for HCC. Ablative strategy has occupied an important position among HCC therapies, based on thermal or non-thermal tumor destruction. RFA, the most common ablation technique applied for patients with HCC, has been developed as a standard treatment, while other ablative techniques have been explored in clinical or preclinical researches.

Thermal ablation, demonstrates similar outcomes as hepatic resection in early HCC (tumors size < 2–3 cm) (9, 10). Other ablation therapies, such as cryoablation, have not established a standard clinic procedure and are therefore less used. In recent years, photodynamic therapy (PDT), photothermal therapy (PTT), magnetic hyperthermia therapy (MHT), and irreversible electroporation (IRE) have shown potential applications in HCC with the prevalence of biomaterials in medicine. The major mechanism of ablative therapies is to induce irreversible thermal (i.e., RFA, MWA, and PTT) or non-thermal tumor destruction (i.e., IRE and PDT) *via* electromagnetic or light energy. This section gives a brief retrospect of traditional ablative therapies for HCC, as well as novel ablative techniques (**Figure 1**), and discusses their immunological effects.

### Clinical Applications

RFA, a standard ablative and first-line therapy for small-sized HCC, is more cost-effective than hepatic resection (10). RFA can achieve tumor necrosis at 375–480 kHz and > 60°C (11). However, traditional monopolar RFA is limited in tumors less than 2–3 cm or near vessels due to heat sink effect, which is also related to recurrence (12). Novel techniques are developed to improve ablation efficacy. No-touch multibipolar RFA can be used to tumors up to 5 cm with similar disease-free survival (DFS) and overall survival (OS) rates compared with resection (13). However, insufficient RFA (iRFA) is one of the major reasons for recurrence after RFA. iRFA could lead to HCC with a more aggressive phenotype, drug resistance and worse prognosis (**Table 1**). The ablative margin assessed by computed tomography (CT) after RFA can be an important predictor of local tumor progression (LTP) and overall recurrence. A study indicated that insufficient ablative margin (<5 mm) was associated with higher rates of LTP and overall recurrence in HCC, but the sensitivity values were only 36.4% and 46.2%, respectively (26). iRFA could promote the proliferation, migration, invasion, epithelial-mesenchymal transition (EMT), and angiogenesis of residual tumors through the transcriptional and epigenetic regulation. Some signaling pathways associated with tumor growth and progression, such as the Akt signaling pathway involved in cellular proliferation, survival and angiogenesis are activated after iRFA (20, 22, 24, 25).

**Table 1:** Mechanisms of phenotype changes after iRFA.

Objects	Phenotypes	Mechanisms	Years	Refs.
HepG2 and MHCC97 cell lines and HCC patient-derived xenograft mouse model	Promoted cell viability and metastasis	m^6^ A-YTHDF1-EGFR axis	2021	(14)
Tumor-associated endothelial cell (TAEC), platelet, HepG2 and SMMC7721 cell lines, and orthotopic tumor mouse model	Enhanced TAEC permeability; activated platelets *in vitro*; and promoted tumor growth, metastasis and endothelial permeability *in vivo*	Upregulation of vascular endothelial-cadherin and ICAM-1	2021	(15)
Hep3B and Huh7 cell lines	Enhanced colony formation, migration, EMT, and angiogenesis; increased resistance to sorafenib	IF1 overexpression and NF-κB activation	2020	(16)
Huh7 cell line, xenograft nude mouse model, and liver metastasis model by tail vein injection	Facilitated cell growth and metastasis *in vitro* and *in vivo*	ceRNA mechanism: ASMTL-AS1/miR-342-3p/NLK/YAP axis	2020	(17)
Huh7 and MHCC97 cell lines	Promoted cell proliferation, migration, invasion, epithelial-mesenchymal transition, and stemness	ceRNA mechanism: GAS6-AS2/miR-3619-5p/ARL2 axis	2020	(18)
HepG2 cell line	Enhanced cell proliferation, colony formation, and migration	c-Met overexpression and MAPK signal pathway activation	2020	(19)
HCCLM3 cell line, xenograft nude mouse model	Induced tumor growth, EMT changes, and metastasis *in vitro* and *in vivo*	Flotillin-1/2 overexpression and Akt/Wnt/β-catenin signaling pathway activation	2019	(20)
HepG2 and SMMC7721 cell lines	Increased cell proliferation, migration, invasion and autophagy *in vitro*	HIF-1α/BNIP3 pathway	2019	(21)
HCCLM3 and HepG2 cell lines, orthotopic nude mouse model	Promoted lung and intrahepatic residual tumor cells *in vivo* and promoted cell migration and invasion *in vitro*	ITGB3 overexpression and FAK/PI3K/AKT signaling pathway activation	2017	(22)
HCCLM3 and HepG2 cell lines, orthotopic nude mouse model	Changed cellular morphology, motility, metastasis, and EMT *in vitro* and *in vivo*	β-catenin signaling activation	2014	(23)
SMMC7721 and Huh7 cell lines, ectopic nude mouse model, and metastasis model by tail vein injection	Enhanced cell proliferation, migration, invasion, and EMT *in vitro*; increased tumor size and lung metastasis *in vivo*	Akt and ERK signaling pathways	2013	(24)
TAEC, HepG2 and HCCLM3 cell lines	Inhibited TAECs proliferation, enhanced TAECs migration and tube formation (angiogenesis); and promoted HCC cell invasiveness	Activation of Akt, ERK1/2 and NF-κB signaling pathways and inhibition of STAT3 signaling pathways	2012	(25)

Several strategies have been used to counter iRFA. One of which is to improve the accuracy of imaging guidance for the specific identification of tumor boundaries, especially with the application of nanotechnology. Jiang and colleagues developed a nanobubble conjugated with colony-stimulating factor 1 receptor (CSF-1R), called NBCSF-1R, for HCC margin detection (27). NBCSF-1R provided a non-invasive effective ultrasound imaging capabilities for evaluating therapy response of RFA through the high specificity targeting of CSF-1R-overexpressing macrophages and HCC tumor margin. Another strategy is the combination therapy for salvage. For instance, sorafenib and IFN-α combined with herbal compound inhibited the EMT of HCC cells after iRFA (28, 29); bevacizumab inhibited the tumor growth and angiogenesis induced by iRFA (30); and CTLA-4 blockade suppressed the growth of residual tumors and improved survival in a subcutaneous murine HCC model (31). Other agents include metformin (32) and hydroxychloroquine (HCQ) (33). However, one study demonstrated that ATPase inhibitory factor 1 (IF1) increased HCC cells’ resistance to sorafenib after iRFA (16). These results indicated that the application of systemic therapy or immunotherapy could cope with the adverse impacts of iRFA but the choice of agents could be limited by iRFA-induced resistance.

MWA could provide higher temperature with expanded ablation zone and shorter ablation time because of its higher frequency (900–2,450 MHz) (11). A recent study showed that MWA provided more excellent tumor control than RFA for patients with perivascular HCC (34, 35). In addition, a meta-analysis of randomized-controlled trials demonstrated that MWA seemed to benefit disease-free survivals at 5 years compared with RFA (36). New microwave thermosphere ablation (MTA) may provide a safer and more effective ablation with shorter time than RFA with the developments of novel MWA systems (37).

Cryoablation is also a thermal technique that could be more effective and safer for tumors not suitable for RFA or MWA, such as perivascular HCC. The goal of cryoablation is to destroy tumor tissue by alternating freezing and thawing on the basis of the Joule-Thomson effect, which benefits low risk vascular complications (38). Moreover, a multicenter randomized controlled trial demonstrated that cryoablation achieved lower local tumor progression than RFA with similar OS and DFS rates (39). IRE is a non-thermal ablative technique that mediates cell damage by changing cell permeability and cellular homeostasis, which lead to cell death (40). Although IRE is a relatively new technique and few clinical studies have been conducted, its safety and efficacy have been proven (41, 42). Similar to cryoablation, one of advantages of IRE is that this technique can be used for tumors not suitable for RFA or MWA, such as perivascular HCC (43).

In a word, ablation take an indispensable place in the clinical treatment of HCC. A series of new techniques have been developed to improve the ablation efficacy and zone to benefit more patients. However, these technologies are image-guided, and their efficacy is closely related to the skills of operators to some extent. This factor is a major barrier to application and an interfering factor that is difficult to eliminate in comparative studies.

### Emerging Ablative Strategies

Phototherapy (e.g., PDT and PTT) is an emerging and prospective cancer therapeutic strategy. Phototherapy kills cancer cells through photochemical or photophysical effects to achieve therapeutic effects. Various photosensitizers (PSs), such as porphyrin-based PDT (44), 5-aminolaevulinic acid-PDT (45) and Radachlorin-PDT (46), could be applied for HCC. However, several factors need to be improved before these methods could be clinically used. First, light (laser) is one of the most indispensable elements in PDT and PTT, on which the therapeutic effect mostly depends. PSs and photothermal agents can be activated only when the light wavelength is in a specific range, known as therapeutic window. Moreover, light wavelength also determines the depth of tissue penetration, which limits percutaneous application of phototherapy to tumors in abdominal parenchymal organs, especially in deep parts. The rapid development of endoscopic techniques and biomedical materials gave rise to strategies to overcome the depth dependence. For example, Li et al. reported laparoscopic-assisted photothermal ablation method based on superparamagnetic iron oxide (SPIO) and new indocyanine green (ICG), called IR820@PEG-SPIO (47). More surprisingly, IR820@PEG-SPIO completely ablated orthotopic liver cancer in nude mice model, as well as detect early-stage HCC (diameter < 2 mm) *via* fluorescence, photoacoustic and magnetic resonance (MR) imaging. Compared with visible light, near infrared (NIR) light and X-Ray can provide deeper penetration (48–51). Besides, MHT, an alternative strategy, has been proposed to further overcome the limitation of penetration depth. Qian and colleagues developed a ferrimagnetic silk fibroin hydrogel (FSH) and demonstrated that FSH-mediated MHT, without depth limitation, could be more suitable for treating liver tumors compared with traditional PTT (52).

Nanoplatforms have stood out because they have improved therapeutic effects and reduced adverse effects, provide precise operation with optimized imaging guidance, and combine therapeutic strategies for synergistic anti-tumor effects. Zhu et al. designed a nanoparticle (ZnPc/SFB@BSA) that combined PDT, PTT and sorafenib with increased efficacy and decreased side effects of sorafenib (53). Jin and colleagues reported another nanoparticle loaded with sorafenib/indocyanine for PDT/PTT/chemotherapy, which could provide synergistic effects against HCC (54). Liu’s group has been devoted to designing different nanoplatforms for combined phototherapy/chemotherapy by aptamer (TLS11a) modification to enhance HCC-specific targeting (55–57). Nanoplatforms may provide more detailed and comprehensive information about tumor size, anatomical structure, and location and realize precise theranostic guidance by applying dual- or multimodal images that integrate optical and traditional medical images (e.g., CT and MR images). For instance, Qi et al. synthesized a NIR-II photoacoustic (PA) CT imaging-guided nanoagent for HCC theranostic strategy, called Pt@PDA-c (58). Pt@PDA-c had deep tissue penetration and high resolution, which provided accurate location of deep (~4 mm intraabdominal depth) and small (diameter < 5 mm) HCC lesions. Moreover, Pt@PDA-c-mediated PTT could eliminated HCC without recurrence under the guidance of real-time PACT.

### Immunological Effects of Ablation

Ablation has long been considered a local treatment. However, growing evidence shows that ablation does more than physically eliminating tumors; it can also play a considerable role in distant lesions through immune effects, also known as abscopal effect. Changes in circulating immune cells/factors and tumor immune microenvironments have been explored by analyzing peripheral blood and tumor models. In 2005, Michael Geissler and colleagues found that local tumor ablation (percutaneous ethanol injection [PEI]/RFA) increased HCC immunogenicity in patients thus to promote endogenous adjuvants release and dendritic cell (DC) activation (59). Besides, RFA induced systemic immune variation in innate immune cells (including natural killer (NK) cells and plasmacytoid DCs) and adaptive immune cells (including tumor-specific T cells, antigen-presenting cells [APCs] and CD8 central memory T cells) (60–62). *De novo* or enhanced tumor-specific immune responses could be observed in patients with HCC after MWA (63). Wu and colleagues observed that neutrophil, monocyte and NK cell were increased to induce innate immune response and immunosuppressive lymphocyte was decreased in patients with HCC post-IRE (64). Moreover, their results indicated an ideal treatment window for immunotherapy (3–14 days post-IRE) to further control tumor recurrence and metastasis. Moreover, the expression of immune checkpoints (programmed cell death protein-1 [PD-1] and PD-1 cognate ligand [PD-L1]), which are associated with HCC tumor size, blood vessel invasion, and BCLC staging, can be downregulated by cryoablation but upregulated at recurrence (65).

The results observed in patients have also been further validated in various animal models. RFA increased CD8+ T cells, memory CD8+ T cells, and DCs and decreased regulatory T (Treg) cells in a unique murine model developed through a combination of intrasplenic inoculated oncogenic hepatocytes and carbon tetrachloride (66). Dai et al. reported that IRE could increase anti-tumor CD8+ T cells to prevent local tumor regrowth and distant metastasis and decrease immunosuppressive Treg and PD-1+ T cells in C57BL/6J mouse model bearing subcutaneous H22 hepatoma (67). Fong’s group demonstrated that IRE induced tumor antigens and facilitated granulocyte macrophage colony-stimulating factor plasmid transfer to achieve local and systemic anti-tumor responses in Yorkshire pig models (68, 69). Similarly, in other solid tumors, RFA can not only reduce the proportions of immunosuppressive cells (including Treg cells, tumor-associated macrophages and neutrophils), but increase the T cell infiltration as well as expression of the immune checkpoints (PD-1/PD-L1 and lymphocyte-activation gene 3 [LAG3]) in RFA-treated tumors and distant non-RFA tumors (70, 71). Moreover, serum and intra-tumoral cytokines, such as IFN-γ, IL-1α/β, IL-2/6/8/10, and TNFα/β, were also increased or activated (64, 72–75).

**Figure 2:**
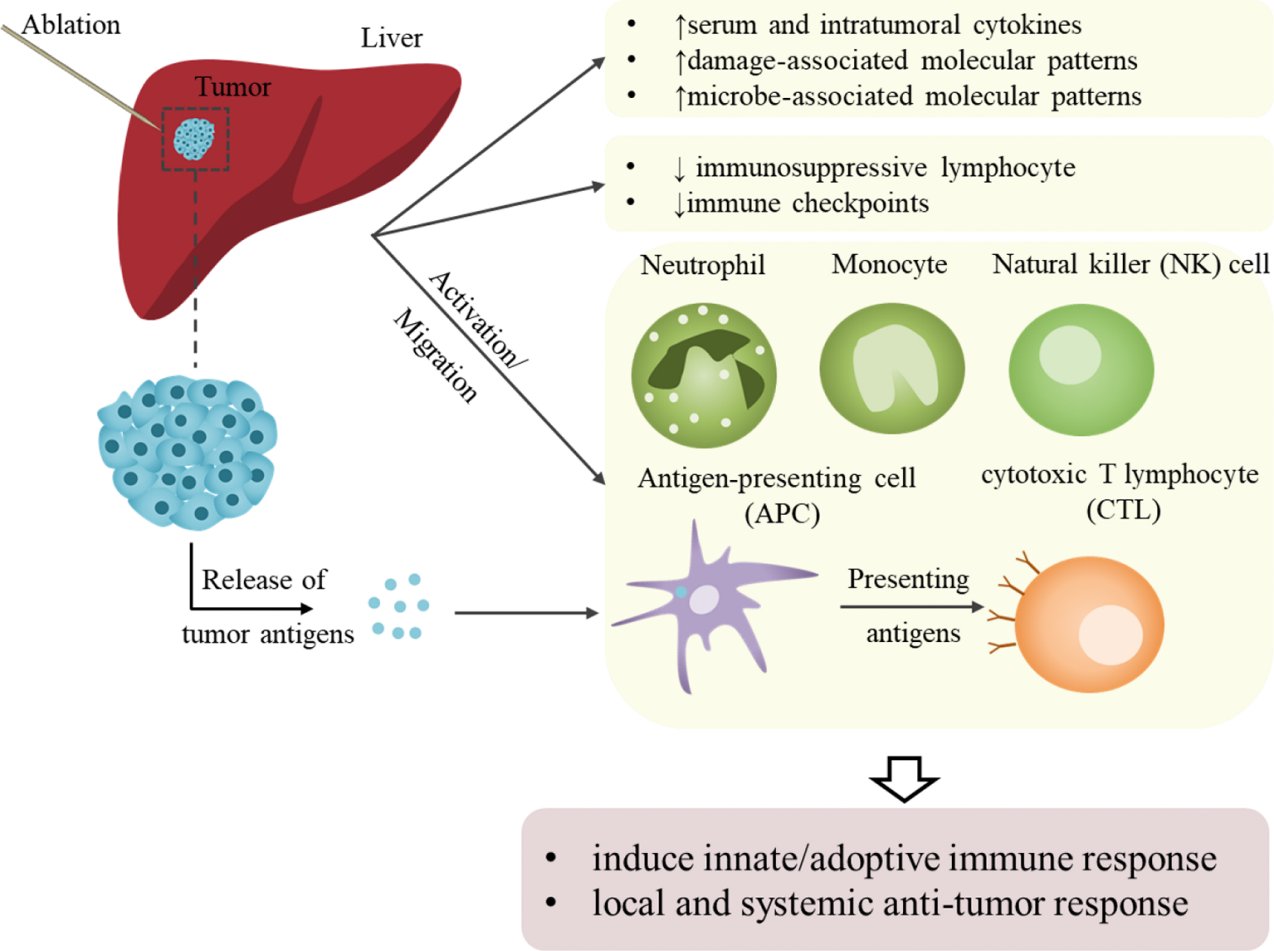
Schematic representation of ablation-induced immunological effects on HCC. Ablation assists local and systemic antitumor responses by activating antitumor immunity and suppressing immunosuppressive effects. On the one hand, the activation of or increase in innate immune cells and cytokines that kill tumor cells achieves non-specific tumor killing. The activation of or increase in adoptive immune cells and the release of tumor-associated or tumor-specific antigens mediates specific anti-tumor immunity. However, these immune effects brought by local ablation are relatively weak and could not meet the requirement needed to sustain anti-tumor effects and prevent recurrence.

Increasing evidence have proved that ablation therapy could activate systemic anti-tumor immunological effects and inhibit immunosuppressive effects (**Figure 2**). However, RFA could also increase PD-1/PD-L1 expression, which was repressed by sunitinib with activation of immune response (66). This effect may facilitate checkpoint inhibitor therapy by constructing an immune-supportive microenvironment. Thus, combining ablation with immunotherapy is rational to achieve augmented and longer anti-tumor effects and prevent HCC progression with improved outcomes.

## Immunotherapy

The 5-year recurrence rates of early HCC with operation or ablation are as high as 70% (6). A retrospective study found that 64 of 103 patients with early/intermediate HCC who received RFA experienced recurrence (76). In addition to the pathophysiological characteristics of the HCC, incomplete treatment response results in the high post-operative recurrence rate, which negatively affects long-term survival. In a meta-analysis reviewing the recurrence rate of HCC after RFA over a ten-year period, the size, number, and location of tumors are partly responsible for incomplete treatment response, limiting the application of RFA in the early 2000s (77). With the introduction of multiple treatment modalities, such as RFA + PEI/TACE, these limitations have been broken and post-recurrence rates have been significantly reduced. However, to complicate matters further, recurrent tumors may be more aggressive (23, 78–80). Thus, adjuvant systemic therapy is taken in consideration. Sorafenib, a multi-tyrosine kinase inhibitor (TKI), has considerably improved the survival of patients with advanced HCC, whereas chemotherapy does not (81). Other emerging TKI drugs, including lenvatinib, regorafenib, cabozantinib and donafenib have been proved to improve the survival benefit of patients with advanced HCC (82–86). However, sorafenib, as an adjuvant therapy for HCC after resection or ablation, did not improved recurrence-free survival (RFS) (82). Furthermore, a phase III STORM trial established a predictive 146-gene signature, which comprised some genes involved in immune-related processes; however, the tested biomarkers and reported prognostic gene signatures lacked value in predicting adjuvant sorafenib on RFS (87). Surprisingly, iodine (131I)-labeled metuximab, an immunotherapeutic agent, proved to benefit RFS of post-operative or post-ablative patients with HCC, in particularly those with CD147+ (88, 89).

**Figure 3:**
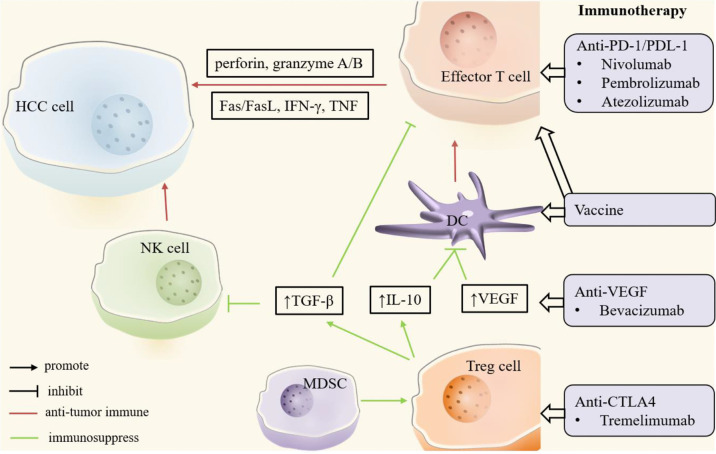
Key players in HCC immune microenvironment. In the HCC microenvironment, natural killer (NK) cells, dendritic cells (DCs) and effector T cells mainly play an anti-tumor immune role (red). Regulatory T (Treg) cells and myeloid-derived suppressor cells (MDSCs) promote tumor immune escape or drug resistance through immunosuppressive effects (green). In addition, tumor growth factor-β (TGF-β), interleukin-10 (IL-10) and other cytokines play an important role in tumor immunity. Immunotherapy enhances anti-tumor immunity or suppresses immunosuppression by targeting these critical cells and molecules. CTLA4, cytotoxic T lymphocyte-associated antigen 4; DC, dendritic cell; FasL, Fas ligand; HCC, hepatocellular carcinoma; IFN-γ, interferon-γ; IL-10, interleukin-10; MDSC, myeloid-derived suppressor cell; NK, natural killer; PD-1, programmed cell death protein-1; PDL-1, programmed cell death protein ligand -1; TGF-β, tumor growth factor-β; Treg, regulatory T; VEGF, vascular endothelial growth factor.

The immune system plays a critical role in HCC, particularly in the HCC development and progression, as well as the treatment response or tolerance (**Figure 3**). Bruno et al. elaborated the HCC immune microenvironment (e.g., antigens, molecular features, and immune cells), and reviewed HCC immunotherapies including immune checkpoint inhibitor (ICI)-based therapies, as well as others based on adoptive cells and vaccines (90). This section will not dwell on the above; instead, it will give a brief retrospect of the application of immune modulators and the advances in novel immunomodulatory strategies.

In short, the goal of immunomodulatory strategies is to activate anti-tumor immune response and/or suppress immune evasion. Immune checkpoints, the surface receptors expressed on immune system cells, include PD-1, PD-L1, cytotoxic T lymphocyte associated antigen 4 (CTLA4), LAG3, and T cell immunoglobulin and mucin domain containing-3 (TIM3) (91). Overexpressed PD-L1 in HCC cells can promote its binding with PD-1 on effector T cells, resulting in immune escape of tumor cells and apoptosis of T cells, which is conducive to the growth and progression of HCC (92). Overexpression of CTLA4 and TIM3 in Treg cells and overexpression of LAG3 and TIM3 in tumor infiltrating T lymphocytes can prevent the activation of effector T cells, also resulting in immune escape of tumor cells (90). The immune checkpoint is one of the immunosuppressive mechanisms that can help HCC immune escape by binding to corresponding ligands in HCC, which is also the rationale for the therapeutic application of ICIs. Recent clinical trials suggested that ICIs, whether alone or in combination with other agents, had a positive effect in HCC. Nivolumab (anti-PD-1), atezolizumab (anti-PD-L1), and tremelimumab (anti- CTLA4), have been proved to be safe and have effective anti-tumor responses for treating HCC (93–95). Notably, nivolumab and pembrolizumab well tolerated and effective in patients with advanced HCC after sorafenib failure with promising effects on long-term survival (96, 97). Atezolizumab, particularly in combination with bevacizumab (anti-VEGF), has superior performance compared with sorafenib in term of survival outcomes and the life quality of patients with unresectable HCC (98, 99).

Other immunotherapies, including adoptive immunotherapies (AITs) and immunotherapeutic vaccinations, activate anti-tumor immune response. AIT improves anti-tumor immunity by expanding or sensitizing lymphocytes *in vitro* and reinjecting them into patients, and cancer vaccines aim to enhance tumor-specific immune responses that are primarily activated by antigen-presenting cells (e.g., DCs) and produce endogenous TAAs. Although these treatments have not been studied as extensively as ICIs, they are under clinical studies. Clinical trials demonstrated the safety and efficacy of T cell- (100), DC- (101), and activated cytokine-induced killer (CIK) cell- (102) based adoptive immunotherapies, as well as oncolytic virus (103, 104) and peptide (105–107) vaccines for HCC. Glypican-3 (GPC3), a carcinoembryonic antigen ideal for immunotherapy target, has been studied extensively as an anti-tumor vaccine of HCC. Phase I/II clinical trials suggested that GPC3 peptide vaccine is effective in inducing cytotoxic T lymphocyte (CTL) killing cancer cells, reducing RFS, and improving OS, particularly in patients with GPC3-overexpressing HCC (105, 108, 109). An animal experiment demonstrated that the synergistic anti-tumor effects depended on increased GPC3-induced CTL though the combination of PD-1/PD-L1 blockade and GPC3 peptide vaccine (110). Moreover, a series of novel GPC3-targeting vaccine (111, 112) and antibodies (113–115) and cellular immunotherapeutic strategies (116–118) against GPC3 rely on the role of GPC3 in HCC and immunotherapy.

Strategies for enhancing therapeutic effects and monitoring immunotherapies have been developed based on advanced technologies. For instance, Liao et al. successfully applied NIR-II fluorescent imaging to NK cell-based immunotherapy for the real-time quantitative tracking and visualization of the viability of adoptive NK cells *in vivo* (119). The potency of immunotherapies can be enhanced by modification with specific antigens (120–122), mRNA optimization (123) and combination with adjuvants (124, 125).

## Combined Ablative-Immunotherapy

As mentioned in Section 2.3, ablation techniques could induce local and systemic antitumor immune responses, but these responses are relatively weak, and cannot completely control the tumor. This reason explains the high local recurrence rates after treatment. RFA activated tumor-specific T cells, but it could not identify a new grown tumor or a recurrent tumor, which resulted in the tumor immune escape and recurrence in a HCC patient (60). Moreover, only 30% of patients with HCC achieved long-term remission and better DFS, because of the tumor-specific immune responses induced by MWA (63). The facts that the application of a single locoregional therapy has a high recurrence rate and locoregional ablation can induce anti-tumor immune responses, have led to the development of combined ablative and systemic therapy studies for recurrence reduction or treatment, as well as improved survival outcomes. Indeed, the advent of TKIs and immunotherapy have improved the outcomes of patients with HCC. Sorafenib, the most promising candidate for adjuvant chemotherapy, failed in patients with HCC after resection or ablation. Results from STORM trial in 2005 showed that compared with placebo, adjuvant sorafenib did not significantly improve RFS in patients with HCC post resection or ablation (126), which is consistent with the findings of existing randomized trials that showed no survival benefit for HCC patients after ablation with adjuvant sorafenib (127, 128). In addition, a study has shown that vitamin K combined with angiotensin converting enzyme inhibitors can inhibit the cumulative recurrence of HCC after treatment (129). A retrospective study has shown that angiotensin II receptor 1 blockers (sartans) can significantly improve overall survival and recurrence time in HCC patients after RFA (130), while another study have shown that this combination can only improve recurrence time (131). These results suggest that more rigorous randomized clinical trials are needed to verify the efficacy of this combination for HCC. On the other hand, the unsatisfying combinations indicated the emergence of immunotherapy as an adjuvant candidate.

In the VX2 tumor model, the combination of RFA and CpG-oligodeoxynuleotides vaccine prevented tumor progression and improved survival outcomes by enhancing anti-tumor T cell response and cytotoxicity (132). Using the CT26 tumor model, Liu et al. studied the roles of palliative RFA (pRFA) in T-cell immune responses and tumor recurrence, which could be more significant in combination with antibodies (74). Likewise, MWA combined with anti-PD-1/anti-CTLA-4 protected mice from recurrence with improved survival (133).

### Clinical Combination on the Way

**Table 2** reviews the finished clinical trials of the combinations of ablation and immunotherapy. Nivolumab and pembrolizumab, which are PD-1 blockades, received quick approvals as second line therapy for patients with HCC after sorafenib failure on the basis of CheckMate-040 (93) and KEYNOTE-224 (97). A recent proof-of-concept clinical trial suggested that the application of RFA or MWA enhanced the anti-tumor effects and response rates (from 10% to 24%) of nivolumab and pembrolizumab (135). The explanation for synergistic effects may be found in Section 2.3 in this review. In brief, the critical roles of RFA in T cell infiltration/response and PD-1 expression may be one of rationales for combining RFA with PD-1 blockade. Besides, the combination of RFA with tremelimumab (CTLA-4 blockade) have been also explored (134, 135).

**Table 2:** Clinical combinations of ablation and immunotherapy.

Ablation technique	Immunotherapy	Efficacies/Outcomes	Research type	Years	Ref.
RFA	CTLA-4 blockade (tremelimumab)	Accumulation of intratumoral CD8^+^ T cells and reduction of HCV load	Phase II trial	2017	(134)
RFA	CTLA-4 blockade (tremelimumab)	Activation of tumor-specific T cell with decreased T-cell clonality	Correlative study	2019	(135)
RFA	PD-1 blockade (camrelizumab)	improved 1-year RFS and OS of patients with recurrent HCC	propensity score matching analysis	2021	(136)
RFA/MWA	PD-1 blockade (nivolumab/pembrolizumab)	Increased response rate with improved survival in patients with advanced HCC after sorafenib failure	Proof-of-concept clinical trial	2020	(137)
RFA	Adoptive immunotherapy (RAK cell)	Feasibility and safety with no severe adverse events, recurrences or deaths in a 7-month follow-up	–	2010	(138)
RFA	Adoptive immunotherapy (NK/γδT/CIK)	Efficiency and safety with improved progression-free survival (PFS) and survival prognosis, decreased HVC load	Open-label	2014	(139)
RFA	Adoptive immunotherapy (CIK)	Increased RFS and OS	Multicenter, randomized, open-label, phase III trial	2015	(102)
RFA	Adoptive immunotherapy (CIK)	Safety with prolonged RFS	Real-word study	2019	(140)
RFA	Adoptive immunotherapy (OK432-stimulated monocyte-derived DC)	Safety with longer RFS; associated with enhanced TAA-specific T-cell responses	Randomized phase I/II trial	2020	(141)
RFA	Vaccine (DC) + multiple antigen (AFP/GPC3/MAGE-1)	Safety and tolerance	Phase I/IIa trial	2015	(109)
RFA	Vaccine (GPC3 antigen)	Improved 1-year recurrence rates in patients with GPC3-positive HCC	Open-label, single-arm phase II trial	2016	(142)
MWA	Adoptive immunotherapy (DC/CIK/CTL)	Safety with ameliorated peripheral lymphocyte percentage	Phase I trial	2011	(143)
cryoablation	Adoptive immunotherapy (DC-CIK)	Increased OS	Retrospective study	2013	(144)

Various studies have demonstrated the safe and effective to applicate adjuvant adoptive cellular immunotherapies to patients with HCC post-ablation with improved RFS and OS (102, 139–141). For patients with metastatic HCC, the combination of cryoablation and DC-CIK cell immunotherapy also achieved a significantly higher OS (median: 32 months) than cryoablation (median: 17.5 months) and the untreated group (median: 3 months) (144). Moreover, the multiple treatment modality for cryo-immunotherapy could provide better prognosis than the single one.

Notably, Tetsuya Nakatsura’s team found that RFA stood out among other locoregional therapies (including surgical resection and TACE) by referring to GPC3-specific T-cell-mediated immune response for HCC (145). Compared with resection, RFA significantly induced GPC3-specific CTLs, especially in patients with GPC3-overexpressing HCC. Consequently, the phase II study of GPC3 peptide vaccine for adjuvant immunotherapy was carried out, laying a foundation of antitumor efficacy of GPC3 peptide vaccine and induced GPC3-specific CTL (105). Although the combination of resection or RFA with GPC3 peptide vaccine decreased the 1-year recurrence rate (142), whether different local strategies had an impact on the prognosis of the combination treatment remained unclear. However, another randomized phase II study showed that adjuvant immunotherapy with tumor associated antigen (TAA)-pulsed DC vaccine prolonged the RFS of patients with complete remission in non-RFA (including surgical resection, TACE, and PEI) groups compared with those in the RFA group (102). These results suggest that combination strategy benefits patients, but the choice of optimal combinations is thought provoking.

### Springing Synergistic Strategies Based on Nanoplatforms

While the clinical trials are in full force, the combination of ablation and immunotherapy is also attracting the attention of scientists in basic medicine and biomedical materials. The development and application of multi-functional nanoplatforms have enabled synergistic ablative-immunotherapy strategy to flourish, instead of the sequential combination. On the one hand, a nanoplatform can deliver multiple drugs with optimized drug performance and therapeutic efficiency, as well as reduced drug toxicity. On the other hand, nanoplatforms can apply imaging technology to identify and locate tumors, guide ablation procedures, as well as monitor drug responses and therapy efficacy. Moreover, such a combination strategy may maximize the synergistic anti-tumor effects and thus achieve a greater therapeutic efficacy than the mere sum of the parts.

First, nanoplatforms can improve the targeting ability of agents through the innate enhanced permeability and retention effect and modifications with specific targets to enhance the anti-tumor effects (8). A mesoporous silica based nanosystem co-loading ICG and sorafenib, named (ICG+S)@mSiO2, was developed for synergetic PTT/immuno-enhanced therapy (146). (ICG+S)@mSiO2 improved endocytosis of HCC cells and photothermal efficiency. Active targeting deliveries were achieved in SP94-PB-SF-Cy5.5 nanoparticles (NPs) (147) and PCN-ACF-CpG@HA NPs (148) by conjugated with HCC specific targeting peptide (such as SP94) and HA (targeting CD44 receptor-overexpressed HCC cells), respectively. Moreover, SP94-PB-SF-Cy5.5 and PCN-ACF-CpG@HA, in combination with PD-L1 blockade and an immunologic adjuvant (CpG), enhanced the PTT- and PDT-induced weak immunogenic cell death of cancer cells. Similarly, these strategies for enhanced immune responses also applied sonodynamic immunotherapy as recently reported by Tan et al. (149) and Lin et al. (150). Moreover, anti-TGF-β antibody modification is an active targeting strategy that enhances cell endocytosis for improved PTT and an immunotherapeutic strategy for immune activation (151). Besides, ICG/ICG-SF-Gel-based photothermal-immunotherapy inhibited primary and distal tumor growth, with improved survival time with the help of Ganoderma lucidum polysaccharides (GLP) for enhancing the antitumor immunity (152). These combined anti-tumor effects led to the application of TAAs in *in situ* vaccination to eliminate residual and distant lesions, as well as inhibit tumor recurrence and metastasis.

Biomimetic nanotechnology, which integrates advantages of nanoplatform delivery and cellular immunotherapy, provides novel strategies for synergistic ablative immunotherapy. On the one hand, biomimetic nanoplatforms are ideal for targeted drug delivery because of their superior biological characteristics. For instance, Wang et al. developed a photothermal immunotherapy nanoplatform based on synthetic high-density lipoprotein (sHDL) (153). The higher expression of sHDL in HCC cells than in other normal cells of liver facilitates the preferential delivery of agents into the cytosol of HCC cells. Ma and colleagues designed a CAR-T cell membrane-coated mesoporous silica NP, which specifically recognized GPC3+ HCC cells (154). On the other hand, a programmable therapeutic strategy based on engineered immune cells provide a possibility for the synergy of ablation and cellular immunotherapy. Zhang et al. constructed an artificial engineered NK cell decorated with TLS11a (a HCC-specific targeting aptamer) for photothermal immunotherapy (56).

## Discussion

The development of science and technology and the deepening of researches on HCC have promoted vigorous developments of treatment strategies for HCC, including the local treatment represented by the clinical standard treatment (RFA) and the emerging phototherapy, and the systematic treatment represented by sorafenib and immune blockers. However, monotherapies have shown some limitations. RFA is a first-line ablative therapy with established technical standard for patients with HCC. However, over 30% of patients suffer from recurrence or metastasis after iRFA (27). The solutions to the problems after iRFA include two aspects: improving the efficiency of RFA and applying combination therapy. The former can be solved well with the development of imaging technology based on nanomaterials, but the process from new drug development to clinical application is long and slow. The latter provides a salvage alternative for residual tumors, but the choice of drugs is thought provoking because of possible drug resistance after iRFA. Moreover, high-quality evidence-based medicine are lacking to support these solutions.

In comparison, increasing evidence support combination therapy. Thus, the combination of ablation and immunotherapy is rationale. On the one hand, ablation can promote anti-tumor immune responses. However, these responses are not strong enough to completely control tumors. On the other hand, the addition of immunotherapy may synergistically amplify the anti-tumor immune effect. The application of nanotechnology and nanomaterials in ablative immunotherapy strengthens the combination; enhances therapeutic effects by improving the physical, chemical, and physiological properties of agents; and achieves a synergistic effect through theranostic nanoplatforms.

Of course, many controversies and challenges need to be resolved. How to develop individualized treatment strategies to obtain the best treatment effect needs to be taken into consideration in clinical research. First, most clinical trials of ablative immunotherapy apply adjuvant immunotherapy after ablation. The frequency of ablation and the optimal time of immunotherapy application need to be specified. For example, a study showed that the ideal time window for immunotherapy after IRE is 3-14 days post-ablation (64). Another study suggested that the frequency of cryoablation is related to prognosis (144). Second, the expression difference of specific genes, such as GPC3 (105, 108, 109), in some patients with HCC leads to different immunotherapy responses and outcomes. Third, the combinations of ablative immunotherapy are diverse. Although some studies demonstrated that ablative immunotherapy provides better outcomes than single ablation or immunotherapy, whether different combinations have differences is unknown. In addition, more multicenter, randomized clinical trials with large samples are needed to confirm the benefits of the ablative immunotherapy. With regard to basic researches, the animal models used in ablative therapy, especially phototherapy, and subcutaneous tumor transplantation model are not suitable because the penetration depth of such techniques is limited. Moreover, tumors in solid organs such as liver, are difficult to reach by percutaneous or laparoscopic ablative techniques, unless the tumor is on the surface of the organ. The development of new drugs based on nanomaterials (such as NIR/X-Ray activated PSs and photothermal drugs) and novel technologies (such as SDT), has been devoted to address these problems. Of course, the success of these advances in cell and animal levels is still a long way from clinical applications. Nonetheless, ablative immunotherapy is expected to gain a place in HCC therapy and benefit patients in the near future.

## Author Contributions

KW and CJ contributed to conceive and design the study. CW, KW, and JM performed the article searching. HJ, WL, and YZ extracted the data. CW and KW wrote the manuscript. CJ and JM supervised the manuscript. All authors contributed to the article and approved the submitted version.

## Funding

This project is supported by the grants from Zhejiang Province Public Welfare Technology Application Research Project (No. LGF21H160022), Natural Science Foundation of Zhejiang Province (No. LQ22H160055), Science and Technology Plan Project of Taizhou (No. 21ywb26 and 21ywb29), Medical Science and Technology Project of Zhejiang Province (No. 2017KY711), and Project of Taizhou University (No.2018PY057).

## Conflict of Interest

The authors declare that the research was conducted in the absence of any commercial or financial relationships that could be construed as a potential conflict of interest.

## Publisher’s Note

All claims expressed in this article are solely those of the authors and do not necessarily represent those of their affiliated organizations, or those of the publisher, the editors and the reviewers. Any product that may be evaluated in this article, or claim that may be made by its manufacturer, is not guaranteed or endorsed by the publisher.
